# A Wireless Smart Adhesive Integrated with a Thin-Film Stretchable Inverted-F Antenna

**DOI:** 10.3390/s24227155

**Published:** 2024-11-07

**Authors:** Ashok Chhetry, Hodam Kim, Yun Soung Kim

**Affiliations:** 1 BioMedical Engineering and Imaging Institute, Icahn School of Medicine at Mount Sinai, New York, NY 10029, USA; ashok.chhetry@mssm.edu (A.C.); hodam.kim@mssm.edu (H.K.); 2 Department of Diagnostic, Molecular and Interventional Radiology, Icahn School of Medicine at Mount Sinai, New York, NY 10029, USA

**Keywords:** wearables, stretchable electronics, body area network, inverted-F antenna, digital health, thin-film electronics

## Abstract

In recent years, skin-mounted devices have gained prominence in personal wellness and remote patient care. However, the rigid components of many wearables often cause discomfort due to their mechanical mismatch with the skin. To address this, we extend the use of the solderable stretchable sensing system (S4) to develop a wireless skin temperature-sensing smart adhesive. This work introduces two novel types of progress in wearables: the first demonstration of Bluetooth-integration and development of a thin-film-based stretchable inverted-F antenna (SIFA). Characterized through RF simulations, vector network analysis under deformation, and anechoic chamber tests, SIFA demonstrated potential as a low-profile, on-body Bluetooth antenna with a resonant frequency of 2.45 GHz that helps S4 retain its thin overall profile. The final S4 system achieved high correlation (R = 0.95, *p* < 0.001, mean standard error = 0.04 °C) with commercial sensors during daily activities. These findings suggest that S4-based smart adhesives integrated with SIFAs could offer a promising platform for comfortable, efficient, and functional skin-integrated wearables, supporting a range of health monitoring applications.

## 1. Introduction

Continuous health monitoring has become a standard practice in both personal care and clinical settings, driven by the miniaturization of electronic devices and advancements in high-speed wireless communication technologies. These devices come in various forms—such as wristbands and socks—and are capable of tracking a range of physiological metrics, including heart rate (HR), respiration rate (RR), blood pressure (BP), blood oxygenation, blood glucose levels, electrocardiograms (ECGs), electroencephalograms, electrodermal activity, posture, gait, and more [1,2,3]. The data collected by these sensors can be instantly transmitted to and displayed on smartphones or tablets, empowering users and caregivers to make informed decisions in real time. Since wearable devices are designed to be worn during various activities, it is essential for them to maintain robust yet intimate contact with the body. The ongoing miniaturization of printed circuit board (PCB) technology and adoption of flexible PCBs (fPCBs) in wearable manufacturing have paved the way for the broader use of patch-type wearables in long-term health monitoring, such as cardiac-event monitoring patches [4] and 14-day continuous glucose monitors (CGMs) [5]. These devices integrate seamlessly into daily life, offering both mechanical durability and functional reliability by housing the electronic components in a rigid or semi-rigid module while allowing an intermediate layer, typically made of hydrogels, to interface with the skin. However, their limitation also lies within this construction. Hydrogels, despite their advantages, present challenges for users with sensitive skin, as long-term and pressurized exposure can cause skin irritation, redness, and dermatitis [6]. Therefore, for users with sensitive skin, typical patch-type wearables could be of a significant concern. Moreover, hydrogels can dry over extended periods and alter the measured data, a disadvantage that should be accounted for in applications requiring consistency in the signal qualities [7]. Several research studies sought to overcome these issues by employing soft dry composite materials (e.g., conductive elastomers) as the skin-contacting electrodes [8,9] as well as the interconnecting traces [10,11]. However, due to fundamental challenges, such as the trade-off in the materials’ adhesiveness and conductivity as well as the instability of the electrical properties as interconnects, soft composite materials have mostly remained as laboratory prototypes or as partially constructed proof-of-concept systems that rely on wired connections. As an alternative approach, a group of researchers demonstrated that thin-film (thickness of ~10 µm or less) metal layers patterned in stretchable formats could serve as fully dry, skin-conformal electrodes. Since the first notable publication on such a sensor system in 2011, referred to as epidermal electronics [12], several systems based on thin-film electrodes have emerged [13,14,15]. However, these systems suffered challenges arising from the drastic mismatch between the fragile thin-film mechanics and ruggedness required by the real-world environment. In 2017, Kim et al. proposed a solution to the problem faced by the thin-film systems by embedding thin, stretchable circuits within medical adhesives, creating a wearable system that retained both thin-film conformality and environmental protection [16]. Notably, the solderable stretchable sensing system (S4) exhibited exceptional solderability for various commercial off-the-shelf chip packages, including a 68-ball Wafer-Level Chip-Scale Package (WLCSP) and compatibility for the industry standard reflow soldering process, suggesting the possibility to retain the conformal electrode-to-skin interface while maintaining system ruggedness and modern electronic functionalities. Table 1 provides an overview of existing soft wearable systems, categorized based on their design approaches for interfacing electronics with the skin.

This paper extends the capabilities of the S4 platform by implementing a fully packaged, Bluetooth-enabled version deployed in free-living conditions. While many prior studies have explored wireless physiological monitoring with stretchable electronics, the presented S4 system reports novel progress in patch-type wearables as the first Bluetooth-capable thin-film electronics to be directly mounted on the skin. Another notable innovation introduced in this article is the integration of a stretchable inverted-F antenna (SIFA), which replaces bulkier chip antennas. This advancement is poised to capture the interest of researchers focusing on thin, conformal antennas for establishing wireless communication between wearable devices and external receivers. As outlined in Table 2, various research groups have previously demonstrated the feasibility of designing thin and compact antennas suitable for wearable applications, using materials such as rigid film, flexible film, liquid metal, and stretchable film. Despite these efforts, including the works of Awan et al. [17] and Hussain et al. [18], there has been a notable gap in showcasing such antennas as functional components of complete wearable systems in real-world scenarios. This shortfall can be attributed to several factors. First, antennas that offer only bendability (and not stretchability) are incompatible with on-body applications due to the mechanical mismatch between non-stretchable substrates and the skin, despite their otherwise superior antenna properties. Second, while liquid metals provide excellent conformability when encased in airtight, soft fluidic chambers, they present challenges such as long-term material instability, potential toxicity, and limited scalability, making them impractical for wearable use [19].

To overcome these limitations, we build upon the findings of Zoul et al. [20,21] to deliver the first demonstration of a skin-conformable, film-based antenna incorporated into a fully functional, standalone wireless health monitoring system. Our work includes simulation and experimental results detailing the effects of stretching and bending on radiation patterns and reflection coefficients. We also examine how antenna performance varies with on-body placement in areas such as the hand, abdomen, and forehead and demonstrate the capability to adjust the antenna’s resonant frequency by modifying its length. Ultimately, we showcase a fully integrated, standalone wireless temperature-sensing patch utilizing the S4 platform with SIFA as a Bluetooth antenna, illustrating the potential for continuous, comfortable, and unobtrusive health monitoring in everyday conditions.

**Table 1:** Comparison of patch-type wearable systems to the presented work.

Systems	Refs.	Entire Electronics Mounted Directly on the Skin?	Uses a Thin-Film Stretchable Antenna?	Demonstrate Use Cases in Free-Living Conditions?
Patch-type wearables for medical use	Commercial single-lead ECG patches	No. Encased in a rigid module.	No. Antennas are integrated within the modules.	Yes.
Prototypes based on thin elements directly mounted on the skin	[12,13,14,15,22,23]	No. Rely on an external connection to circuits.	No.	No. Only in controlled settings.
Prototypes based on soft materials	[9,24,25,26]	No. Only the sensor elements contact the skin.	No. Stretchable antennas are based on thick, printed materials or liquid metals.	No. Only in controlled settings.
S4	This work and [16]	Yes.	Yes.	Yes.

**Table 2:** Comparing SIFAs to representative thin, compliant antennas designed for health monitoring applications.

Form Factors	Refs.	Dimensions (w × l × h in mm) ^1^	Demonstrate On-Body Applications?	Integrated as Part of a Standalone Device?	Demonstrate to Use Cases in Free-Living Conditions?
Rigid film	[18]	19 × 21 × 1.6	No	No	No
Flexible film	[17]	28 × 33 × 0.168 ^2^	No	No	No
Flexible film	[27]	83 × 89 × 1.52	No	No	No
Flexible film	[28]	30 × 20 × 0.05	Yes	No	No
Liquid metal (flexible)	[29]	15 × 15 × 0.81	No	No	No
Liquid metal (stretchable)	[30]	6 × 40 × 1.6	No	No	No
Stretchable film	[31]	36 × 36 × 2	Yes	No	No
Stretchable film	[32]	5 × 46 × 0.2, 22 × 25 × 2	Yes	No	No
Stretchable film	This work	26 × 36 × 0.006	Yes	Yes	Yes

^1^ w, l, and h stand for width, length, and thickness of the antenna. ^2^ Thickness was not provided and is estimated based on the indicated use of the RO4835 substrate (Roger Corporation, Chandler, AZ, USA).

## 2. Design, Fabrication, and Characterization of a SIFA-Integrated S4 with Bluetooth Capabilities

### 2.1. Design and Fabrication of Thin-Film Transmission Lines

**Figure 1:**
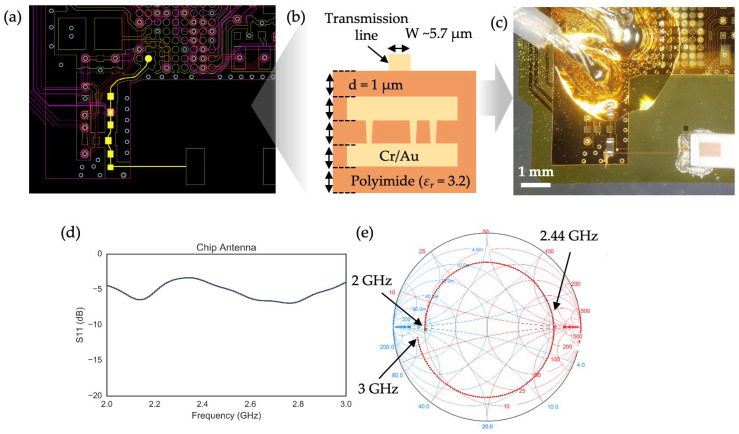
Design and fabrication of a thin-film transmission line: (**a**) CAD drawing defining the board layout and trace outlines for the BLE module; (**b**) illustrative cross-sectional view of the thin-film 2-layer circuit system specifying the layer thicknesses and dielectric constant (*ε_r_*) for the polyimide; (**c**) Fabricated BLE module with a narrow transmission line is connected with a chip antenna. The coaxial cable (top left) is soldered to the RF and ground pins of the circuit and affixed by epoxy; (**d**) return loss (S11) parameter plotted over 2 to 3 GHz range using a vector network analyzer; (**e**) measured return loss data represented as a circle on a Smith chart, the center of which represents a purely resistive impedance of 50 Ω.

As a first step toward developing and demonstrating a Bluetooth-enabled S4 system, we selected a commercially available Programmable System-on-Chip (PSoC) from Cypress Semiconductors (San Jose, CA, USA), featuring integrated Bluetooth Low Energy (BLE) RF functionality. The programmability of the PSoC offered flexibility throughout the design process, while its built-in features—such as the analog-to-digital converter (ADC), operational amplifiers, and memory—reduced the need for additional external components. Most importantly, the chip was available in a compact Wafer-Level Chip-Scale Package (WLCSP) with 68 preformed solder balls using the SAC305 system, a widely used lead-free solder alloy composed of 96.5% tin (Sn), 3.0% silver (Ag), and 0.5% copper (Cu). To examine the preliminary performance of the S4-based BLE module, we designed a two-layer circuit as shown in Figure 1a following the microfabrication techniques and thin-film structures described in [16], along with the board and trace design guidelines provided by the PSoC manufacturer. However, since the standard guidelines are based on the assumption of using the conventional board material (e.g., FR4) and thickness of millimeters, a design modification was needed for the conductive trace, referred as a transmission line, that transfers RF signals between the RF pin of the PSoC and the chip antenna (Figure 1b). To do this, we utilized the simplified design guideline of a microstrip transmission line described by Visser et al. [33]:

(1) $$\frac{W}{d}=\frac{2}{\pi }\left[A-1-\ln\left(2A-1\right)+\frac{\varepsilon_{r}-1}{2\varepsilon_{r}}\left\{\ln\left(A-1\right)+0.39-\frac{0.61}{\varepsilon_{r}}\right\}\right],$$

where *d* is the thickness of the dielectric, *ε_r_* is the relative permittivity of the dielectric, and

$$A=\frac{377\pi }{2Z_{0}\sqrt{\varepsilon_{r}}}$$

Since most modern RF electronics operating at ~2.4 GHz, including the PSoC and our selected chip antenna, have a characteristic impedance of 50 Ω, we can determine that the required trace width (W) is approximately 5.7 µm, assuming a trace thickness of 1 µm and a dielectric layer with a relative permittivity of 3.2 for the polyimide dielectric (Figure 1b). However, in microfabrication processes—especially those involving photolithography and wet etching—precisely controlling each lithographic step to achieve accurate trace widths is extremely challenging. As a result, it is often necessary to overcompensate for potential feature size losses (e.g., due to over-etching or over-development) by designing the trace wider than required. This overcompensation, however, can lead to traces that are significantly wider than intended, making it difficult to achieve the desired 50 Ω impedance for thin-film transmission lines using lithographic methods alone, particularly in a research lab environment. For example, Figure 1c shows an optical micrograph of a transmission line designed with an intended width of 5.7 µm, but the actual measured width exceeded this value. Consequently, the measured S11 parameter, which represents the reflected power relative to the incident power, indicated significant power loss, as evidenced by the small absolute S11 values in the 2 to 3 GHz range (Figure 1d). The impedance mismatch of the trace was further confirmed by plotting the S11 parameters on a Smith chart, a graphical tool used to visualize complex impedance, where the center of the chart represents the 50 Ω point. The S11 data point at 2.44 GHz was far from the center, indicating substantial power reflection (Figure 1e). Therefore, while matching the 50 Ω characteristic impedance of the antenna trace in the S4 platform is theoretically achievable, consistently controlling the trace width remains a significant technical challenge.

### 2.2. Implementation of a Bluetooth-Enabled S4

**Figure 2:**
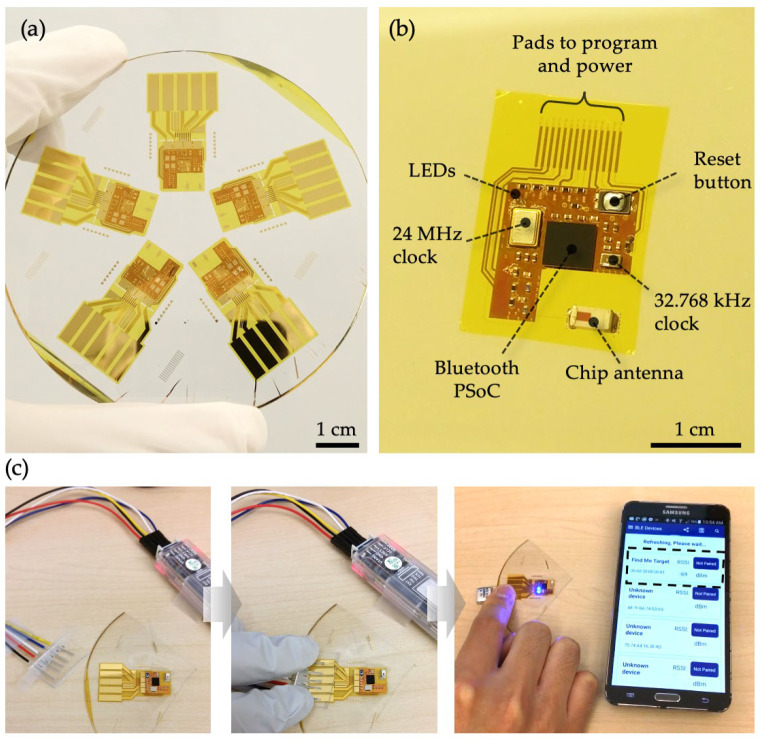
Overall process involved with implementing Bluetooth capabilities with an S4: (**a**) Photograph of a glass wafer containing five S4s designed for a Bluetooth circuit system; (**b**) An S4 assembled with surface mount components necessary for basic Bluetooth functionality; (**c**) Steps involved with confirming the system functionality from positioning of the programming and power wires (left), contacting the wires with the S4 by pressing with figures and a thin PDMS spacer (center), and verification of Bluetooth advertisement using a mobile application while powering the S4 with a small lithium-ion polymer battery. Recorded RSSI value was −69 dBm.

Despite the suboptimal return loss measurements, we assembled the fabricated circuits with surface-mount components to evaluate the overall functionality and integrity of the S4 as a BLE module. Figure 2 illustrates the process, which includes microfabrication, reflow soldering of surface-mount components, firmware programming, and verification of Bluetooth transmission. Using previously described microfabrication techniques and thin-film handling methods, we fabricated S4s designed to accommodate surface-mount chip components required for a basic Bluetooth microcontroller (Figure 2a) [16]. Figure 2b shows the results of the reflow soldering process, which successfully integrated various surface-mount components onto the S4, including the 68-ball WLCSP package, crystal oscillators, a push-button switch for toggling Bluetooth advertisements, a chip antenna, passive components (resistors, capacitors, and inductors) in 0201 packaging, and LED chips. As depicted in Figure 2c, programming and powering the fully assembled S4 were achieved by pressing exposed jumper wire cores against the contact pads using fingers, stabilized by a thin polydimethylsiloxane (PDMS) spacer to hold the wires in place. Once the firmware, based on a BLE protocol, was successfully programmed, a small lithium-ion polymer battery was temporarily connected to power the S4. Finally, a mobile application designed for the Cypress PSoC was used to verify that a mobile phone could detect the BLE advertisement broadcast by the S4. However, the signal strength was weaker, as anticipated by the VNA measurements, with a low Received Signal Strength Indicator value of −69 dBm, even when the S4 was near the mobile phone.

### 2.3. Development of a Stretchable Inverted-F Antenna (SIFA)

#### 2.3.1. Ground Length Optimization

**Figure 3:**
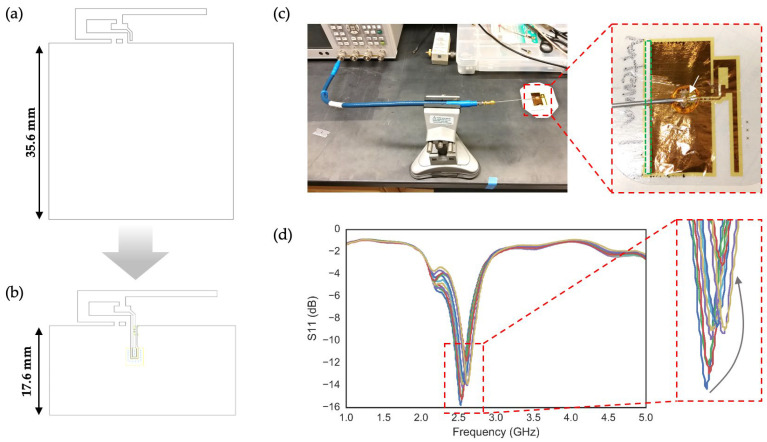
Effects of ground length on IFA properties: (**a**) Reference IFA design adopted in previous works with a ground length of 35.6 mm; (**b**) Reduced ground length (of 17.6 mm) adopted in this work; (**c**) VNA measurement set up showing the flexible SubMiniature version A (SMA) cable held by a clamp holding the Tegaderm-integrated IFA in air. The zoomed inset show the portion removed with a razor blade (green dotted lines) and the soldered coaxial connection to the feed point of the antenna (white arrow); (**d**) Series of S11 measurements during the incremental reduction of the ground length by 1 mm, from 17.6 mm to 11.6 mm. The arrow in the inset indicates the general trend in the shift of the resonant frequency and respective S11 magnitude as measured during the ground length reduction.

The inverted-F antenna (IFA) is a type of monopole, quarter-wave antenna named for its characteristic shape. IFAs are commonly used in cellphones and other wireless devices due to their high efficiency, low cost, and ability to be printed directly onto a PCB. While the standard IFA design follows conventional transmission line theory, Texas Instruments (TI; Dallas, TX, USA) developed a specific IFA design that achieves a 50 Ω characteristic impedance, provided the antenna layout strictly adheres to the reference design (Figure 3a). In previous work, Zoul et al. used this reference design to create a stretchable, flexible, and adhesive-integrated IFA for preliminary on-skin use [20,21]. However, their design had several limitations. First, the IFA allowed only the ground plane to stretch, while the radiating element retained a solid structure. Second, the length of the ground plane was chosen arbitrarily, and its impact on antenna performance was not thoroughly investigated. To address these limitations, we began by optimizing the ground length. We fabricated solid IFAs with a ground plane half the length of that used by Zoul et al. (17.6 mm, Figure 3b) and embedded the IFAs in a medical adhesive (Tegaderm, 3M, St. Paul, MN, USA). The adhesive-embedded IFAs were then connected to a vector network analyzer (VNA; E5071, Agilent Technologies, Santa Clara, CA, USA) using the method described earlier (Figure 3c). Initially, the IFA with half the ground length showed no significant degradation in performance and exhibited a strong S11 parameter of −15.76 dB. We then progressively reduced the ground plane by cutting it perpendicular to its length in 1 mm increments using a razor blade. As shown in the S11 data in Figure 3d, each cut led to a reduction in the magnitude of S11 and a slight increase in the resonant frequency. Based on these results, we concluded that while further reduction in ground length is possible, a stretchable IFA (SIFA) with half the ground plane length is a solid starting point. Smaller ground planes could be considered for applications requiring a smaller device footprint; however, further tuning would be necessary to compensate for the increase in resonant frequency and decrease in S11 magnitude.

#### 2.3.2. Fabrication and Optimization of a SIFA

**Figure 4:**
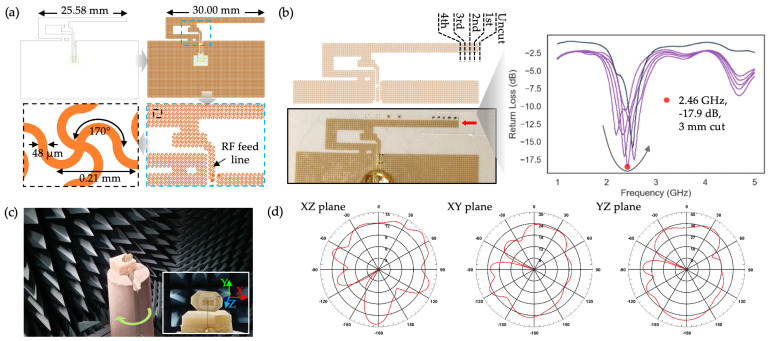
Fabrication and optimization of a SIFA: (**a**) Details of the serpentine mesh design applied to the SIFA with a trace width, arc angle, and mesh spacing of 48 µm, 170°, and 0.21 mm, respectively; (**b**) The radiating element was incrementally shortened from an initial length of 30.00 mm, with each cut reducing its length by 1 mm using a razor blade. Dashed lines in the top left illustration mark the locations of each cut. The bottom left photograph shows the SIFA after the fourth cut, with the red arrow indicating the location of the most recent cut. The plot in the right shows five respective S11 measurements for each length with the red dot indicating the optimal properties measured at the 3rd cutting, equivalent to 27.00 mm. For comparison, S11 data measured with a non-stretchable IFA with the ground length reduced to 17.6 mm is plotted in blue grey; (**c**) The anechoic chamber measurement setup involving the SIFA fixed on a rotating stage, connected to a VNA; (**d**) Experimentally measured SIFA’s 2D radiation patterns in the three planes.

To create a fully stretchable inverted-F antenna (SIFA), we applied the serpentine mesh pattern previously used in the respiration-sensing S4 [16] to the solid IFA design described in Section 2.3.1. As shown in Figure 4a, the mesh consists of a serpentine copper pattern with a trace width of 48 µm and an arc angle of 170°. While similar to the design used by Chang et al., our mesh has a significantly finer spacing of 0.21 mm, compared to 1.65 mm in their study [31]. In a method akin to the ground length optimization, the radiating element of the SIFA was initially extended beyond the reference design to allow for a detailed examination of its RF properties as the length was sequentially reduced by cutting with a razor blade. Consequently, the initial antenna length was set to 30 mm, rather than the standard 25.58 mm. As depicted in Figure 4b, S11 measurements were taken in air, starting with a length of 30.00 mm, and sequentially shortened by 1 mm for a total of four cuts. The resulting S11 data show that the optimal antenna performance, with a maximum S11 value of −17.9 dB and a resonant frequency of 2.45 GHz, was achieved after the third cut, corresponding to an antenna length of 27.00 mm. Compared to the non-mesh solid IFA, which showed a resonant frequency of 2.53 GHz and peak S11 of −15.8 dB with a reduced ground length (plotted in blue grey in Figure 4b), the SIFA with a 27.00 mm radiator length exhibited better performance as a Bluetooth antenna. However, since the solid IFA’s radiator length remained fixed at 25.58 mm, we anticipate that extending its length would result in an S11 trend similar to that observed with the SIFA. To provide a more comprehensive assessment of the antenna’s properties, we also measured the 2D radiation patterns in the XZ, XY, and YZ planes. This was performed by rotating the antenna on a stage and capturing its transmitted power in an anechoic chamber (Figure 4c). As shown in Figure 4d, the radiation patterns, represented as normalized gain in polar coordinates, indicate that the antenna exhibits strong, omnidirectional radiation in the XY and YZ planes, with slightly weaker and less uniform radiation in the XZ plane. Together, the VNA and anechoic chamber measurements confirm that the SIFA, with a radiating element length of 27.00 mm and a ground length of 17.6 mm, is a promising candidate for use as a BLE antenna.

#### 2.3.3. Effects of Bending and Stretching of a SIFA

**Figure 5:**
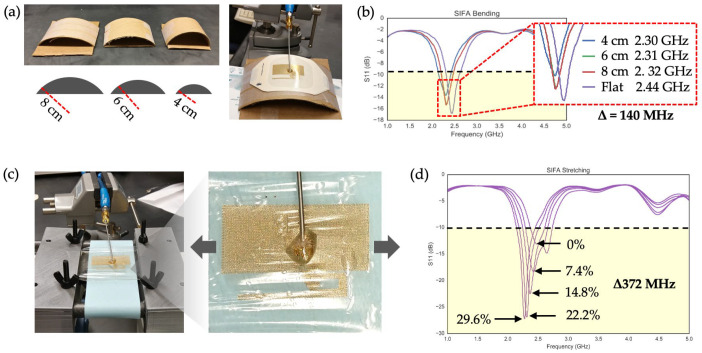
Effects of bending and stretching of a SIFA: (**a**) Paper-based curvatures with bending radii of 8 cm, 6 cm, and 4 cm (left). The experimental setup used to measure S11 from a bent SIFA (right); (**b**) A series of four S11 data measured from a SIFA in 3 different bent states and flat; (**c**) The experimental set up used to measure S11 while biaxially stretching a SIFA shown in high angle (left) and top (right) views; (**d**) A series of five S11 data measured from a SIFA in 4 varying stretched states and unstretched. The total shift in the resonant frequency caused by stretching the SIFA from its unstretched state (0%, resonant freq. = 2.460 GHz) to the final stretched state (29.6%, resonant freq. = 2.268 GHz) is 372 MHz.

Given that the primary use case for the SIFA is in on-body applications, the effects of two modes of deformation—bending and stretching—on the antenna’s performance were investigated. As illustrated in Figure 5a, curved surfaces with three different bending radii (8 cm, 6 cm, and 4 cm) were created by cutting sections of paper cylinders. These radii were selected to simulate body areas with curvature, such as the arms, legs, and forehead. A series of S11 measurements were then performed while bending the SIFA against each curvature, starting with a baseline “flat” measurement. As shown in Figure 5b, decreasing the bending radius resulted in a reduction in the S11 magnitude and a 140 MHz downward shift in the resonant frequency. However, in all cases, the bandwidths—defined by S11 values below −10 dB—sufficiently covered the RF frequency range required for BLE communication.

Since most parts of the human body undergo regular extension and relaxation, the effects of stretching on the SIFA were also evaluated. The antenna was uniaxially stretched along the length of the radiating element, as this direction was expected to have a greater impact than the perpendicular direction (i.e., along the ground length). The antenna was clamped at both ends using a custom-built stretching apparatus (Figure 5c). Figure 5d presents the evolution of the S11 plots as the SIFA was strained up to 30%. A percentage of 30% was chosen as a conservative strain limit, considering that most parts of human skin do not exhibit strain higher than 15% [34]. Despite the shift in the resonant frequency from 2.460 to 2.268 GHz, the bandwidth of interest at all strain levels remained within the −10 dB threshold, indicating the SIFA’s capability to maintain BLE connectivity even when subjected to strains up to 30%. Two noteworthy phenomena were observed. First, the return loss improved with increasing strain, likely due to an increase in the effective ground plane caused by stretching. Additionally, the resonant frequency decreased as the effective length of the radiating element increased, consistent with the inverse relationship between antenna length and resonant frequency.

#### 2.3.4. Effects of On-Body Applications on a SIFA

**Figure 6:**
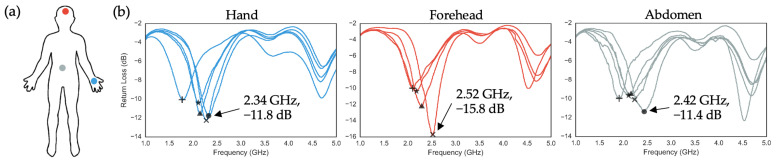
Effects of on-body applications on SIFA properties: (**a**) Three application sites used to measure S11 from SIFAs. Blue, red, and grey represent the hand, forehead, and abdomen, respectively; (**b**) S11 measurements taken from each body location as the radiating element’s length is gradually shortened by 1 mm. Percent of reduction in the radiator’s length is denoted by different symbols. +, ★, ▲, ×, and ● denote 0%, 0.033%, 0.066%, 0.100%, and 0.133% reduction in the length, respectively. Arrows indicate the coordinates of the optimal pairs of S11 and resonant frequency for each experiment.

The human body is known to significantly affect antenna performance, impacting gain, radiation pattern, return loss, and impedance matching due to its ability to scatter and absorb radiated power [35,36,37]. In this section, we present S11 measurements of SIFAs applied to three body locations: the hand, forehead, and abdomen (Figure 6a). Following previous methods, the SIFAs were initially designed with a 30 mm antenna length, which was incrementally shortened by 1 mm using a razor blade for each on-body measurement. As shown in Figure 6b, the number of cuts needed to optimize antenna properties varied by location, based on how close the resonant frequencies were to the Bluetooth range and the magnitude of the S11 values. The optimal antenna characteristics for each location were 2.34 GHz, −11.8 dB (hand); 2.52 GHz, −15.8 dB (forehead); and 2.42 GHz, −11.4 dB (abdomen). The forehead exhibited the greatest sensitivity to length reduction, with the resonant frequency shifting beyond the Bluetooth range after just three cuts (0.100% length reduction). This pronounced response is consistent with previous studies, which indicate that the forehead’s complex tissue structure—characterized by thin skin over bone—contributes to stronger electromagnetic coupling, leading to more significant impedance mismatches and frequency shifts compared to areas with thicker, soft tissue layers. Moreover, the high dielectric constant of the forehead’s tissues further amplifies the impact of small length adjustments on resonant frequency, as shown in similar experimental setups for near-field antennas [38]. In contrast, the hand and abdomen, which have thicker and more uniform soft tissues, demonstrated lower sensitivity, achieving optimal performance after four cuts (0.133% length reduction). This aligns with prior observations that regions with greater soft tissue buffering exhibit more stable resonant behavior, even when subjected to incremental length modifications [39]. These results demonstrate a clear pattern in how antenna properties change with length reduction across different body sites and offer critical insights for optimizing antenna performance in the design of future S4-BLE devices, enabling more effective tuning based on specific on-body applications.

## 3. Axillary Temperature Monitoring with an S4-BLE Device Integrated with a SIFA

**Figure 7:**
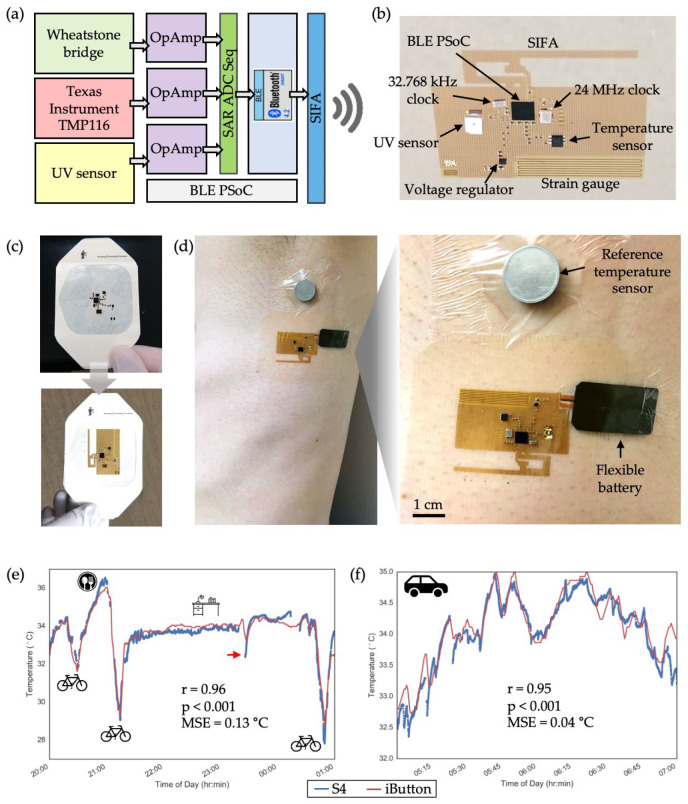
Demonstration of axillary temperature monitoring with a S4-BLE device: (**a**) Sensor data flow and management scheme used in the device. Utility of the Wheatstone bridge circuit and UV sensor is not discussed in this study; (**b**) A S4-BLE embedded with a SIFA assembled with surface mount components; (**c**) Embedding the assembled S4 device in a medical adhesive (Tegaderm). Holes were patterned with a laser to allow relatively thick chip components to pass through the adhesive; (**d**) Tegaderm-integrated S4 attached in the axillary region (left). The zoomed view shows the flexible battery attached to the S4 using an extra piece of Tegaderm as well as the reference temperature sensor (iButton) affixed by a Tegaderm (right); (**e**) Continuous axillary temperature data measured with the S4 and iButton during daily activities. The 5-hour-long temperature data measured in a free-living condition show a highly correlated temperature fluctuations based on the participant’s location (indoor vs. outdoor) and activities (riding a bike, eating, desk work). The red arrow indicates a moment of brief BLE disconnection; (**f**) The 2-hour-long data measured during a driving scenario also shows a high correlation between the two devices. (MSE: mean standard error).

Building on the results from previous sections, we explored the feasibility of integrating a SIFA into an S4-BLE device for continuous health monitoring. To do so, a new circuit design was developed to collect, process, and transmit data from multiple sensors—such as strain, UV exposure, and temperature—using the BLE circuit described in Section 2.2 (Figure 7a). This study focuses on temperature data collected by a digital temperature sensor chip (TMP116, TI, Dallas, TX, USA). As illustrated in Figure 7b, the surface-mount components were assembled using a reflow soldering technique demonstrated by Kim et al., where the soldered S4 remained on a PDMS-coated glass wafer prior to integration with an adhesive film. To embed the S4 into a medical adhesive, a CO_2_ laser was used to selectively ablate Tegaderm, creating openings that aligned with the surface-mount components. This ensured flush contact between the Tegaderm and the S4, allowing the device to be efficiently removed from the wafer (Figure 7c). The adhesive-integrated device was then applied to the axillary region for real-time temperature monitoring, alongside a commercially available temperature logger (iButton, iButtonLink Technology, Whitewater, WI, USA) attached with Tegaderm. To protect the exposed chip components from environmental factors and friction with clothing, a liquid bandage (Nexcare No Sting Liquid Bandage, 3M, St. Paul, MN, USA) was sprayed over the device (Figure 7d). Data collection was performed in two scenarios to simulate various daily activities. In all scenarios, the temperature data sampled at 1 Hz were transmitted to a smartphone placed in the user’s pants pocket. The first scenario spanned five hours, covering indoor and outdoor activities such as cycling, dining, and desk work. Correlation analysis between the S4 and iButton data revealed a strong and statistically significant correlation (r = 0.96, *p* < 0.001) with a mean standard error (MSE) of 0.13 °C (Figure 7e). In the second scenario, which lasted two hours while the user was driving, the analysis (Figure 7f) showed a similarly high level of agreement (r = 0.95, p < 0.001, MSE = 0.04 °C). In both cases, the S4 captured more detailed temperature fluctuations, likely due to its higher sampling rate (1 Hz vs. 0.0167 Hz), higher resolution (0.0078 °C vs. 0.125 °C), and closer proximity to the skin. Apart from one instance of Bluetooth disconnection during the five-hour test, caused by the user moving more than 10 m away from the mobile phone, the results demonstrate the potential of the S4-BLE device with a SIFA as a viable health monitoring platform. The system effectively integrates with daily routines, providing continuous monitoring with minimal disruption to the user’s lifestyle.

## 4. Discussion

In the era of digital medicine enabled by wearable sensors and devices, the demand for electronic sensor platforms that integrate seamlessly with human life, both mechanically and in terms of comfort, is becoming increasingly important. While wrist-worn health monitoring devices dominate the wearables market, patch-type wearables present unique advantages by adhering directly to various body sites of interest, offering novel physiological data that cannot be easily captured from the wrist.

Most current patch-type wearables use a modular form factor, where the electronic module is housed in a small, rigid enclosure that can be detached from a disposable patch layer. While this design allows for the recovery and reuse of the electronics, the compact footprint of the module often increases bulk, causing discomfort, especially in regions that experience frequent movement, such as the knees, elbows, hands, and face. In scenarios, where sensor placement near these areas is crucial, the S4 wearable platform offers an attractive alternative. Its minimal physical presence and highly compliant mechanical properties embody the concept of a wireless electronic medical adhesive.

When comparing the SIFA-integrated system to traditional chip antenna systems, several considerations arise. Chip antennas in wearable devices generally exhibit a gain of 0 to 2.5 dBi, a return loss of less than −10 dB, and transmission distances of 5 to 30 m, depending on the environment and device configuration [40,41,42]. While our study did not focus on direct gain measurements or extended transmission tests, the SIFA system’s flexible, ultra-thin design offers potential advantages in user comfort and mechanical adaptability, which are critical for prolonged on-body applications. The SIFA system, by conforming closely to the skin, minimizes bulk and improves mechanical integration compared to the rigid structures of electronic modules and chip antennas commonly found in typical wearables, which are often less suitable for long-term use, especially on high-motion body regions. Furthermore, the SIFA’s radiating element could potentially enhance radiation efficiency and impedance matching across non-hair-bearing body sites by reducing energy loss from body absorption. In contrast, chip antennas are more likely to suffer from reduced performance when placed directly on the skin due to higher dielectric loading and body coupling effects, which limit their efficiency and transmission capabilities [41,42].

While the optimization of SIFA’s wireless performance, such as gain and radiation efficiency, as well as the investigations into the specific absorption rate and the effects of other bodily factors like sweat, remains an area for future research, the results presented here highlight the platform’s versatility across a wide range of non-hair-bearing body sites. These advancements pave the way for next-generation digital healthcare solutions centered on a wireless adhesive electronic sensor platform. The adaptability of the SIFA system to conform closely to the body’s contours, combined with its low-profile design, offers clear advantages in not only human studies, but possibly for small-animal studies [43,44], where traditional, rigid devices often face limitations.

The broader implications of the S4 platform’s design extend beyond individual metrics such as gain or return loss. Its innovative structure not only improves wearability and comfort but also supports applications where long-term, stable sensor placement is critical. Potential applications include multi-lead ECG patches for high-fidelity, post-surgical cardiac monitoring, large-area EMG patches for diagnosing muscular disorders and tracking recovery, and multifunctional abdomen patches for fetal health monitoring. By prioritizing mechanical flexibility and skin integration, the SIFA system represents a shift toward more user-friendly, adaptable wearables in the field of digital medicine.

In summary, although this study did not conduct direct comparisons with chip antenna systems for parameters such as gain or transmission distance, the findings support the SIFA system’s potential to address key challenges associated with traditional Bluetooth-based wearable devices. The combination of simulation results, experimental outcomes, and literature-based comparisons suggests that the SIFA-integrated S4 could be a novel patch-type wearable platform that retains both the fully dry and direct electrode–skin interface and mechanical reliability necessary for deployment in free-living conditions.

## 5. Conclusions

This study presents significant advancements in the development of skin-integrated, wireless health monitoring systems by leveraging the S4 platform and incorporating a novel stretchable inverted-F antenna (SIFA). The results highlight the SIFA’s potential as a thin, conformal, and mechanically adaptable Bluetooth antenna that enhances the overall functionality and comfort of the S4 system. Our comprehensive analysis, which included RF simulations, vector network analysis, and real-world testing, demonstrated that the SIFA maintains reliable performance under various mechanical deformations such as bending and stretching, as well as across different body sites.

The fully integrated S4-BLE system, featuring the SIFA, achieved robust temperature monitoring during daily activities with high accuracy and strong correlation to commercial sensors, proving its capability for continuous, real-world health tracking. This demonstrates that integrating thin-film, stretchable antennas with advanced wearable platforms can effectively overcome limitations posed by conventional chip antennas, such as mechanical rigidity and bulk, and enables seamless, long-term wear.

## Data Availability

The data presented in this study are available on request from the corresponding author.
